# Goji Berry (*Lycium Barbarum* L.) Carotenoids Enrichment through ‘Green’ Extraction Method Improves Oxidative Stability and Maintains Fatty Acids of Yak Ghee with Microwave Heating and Storage

**DOI:** 10.3390/foods11030369

**Published:** 2022-01-27

**Authors:** Anita Nkansah Agyare, Chang Hong An, Qi Liang

**Affiliations:** Functional Dairy Products Engineering Laboratory of Gansu Province, College of Food Science and Engineering, Gansu Agricultural University, Anning District, Lanzhou 730070, China; anniejay96@gmail.com (A.N.A.); Changhongan1@outlook.com (C.H.A.)

**Keywords:** yak dairy, dairy processing, edible oils, ultrasonic-assisted extraction, accelerated storage, radical scavenging activity, high shear dispersion, color

## Abstract

As the oxidation of yak ghee is inevitable and as consumer demand for natural products continues to increase, this study aimed to enrich yak ghee with goji berry carotenoids by means of green solvent extraction and determined changes in the oxidative stability and fatty acid profiles of yak ghees during microwave heating (MW-heating) and accelerated storage. An enriched ghee (GG0) was prepared by high shear dispersion and ultrasound-assisted extraction, while a control ghee (FG0) was prepared by heating and filtration; both ghees were stored at 65 °C for 30 days and were microwave-heated (MW-heating) at 180 °C (15 and 30 min) and 200 °C for 30 min. The results showed that the carotenoid enrichment increased the oxidative stability of yak ghee during MW-heating and storage. The initial CLA and PUFA values of GG0 were not significantly different from those of FG0; SFA increased, and MUFA and TFA decreased. There was a faster rate of UFA loss and an increase in SFA and TFA in FG0 during MW-heating and storage. This indicated a protective effect of carotenoid enrichment on yak ghee. Therefore, the findings in this study support the use of goji berry carotenoids as a natural colorant and antioxidant in yak ghee. This study provides vital information for dairy processors and marketers.

## 1. Introduction

Yak ghee is the clear golden-colored oil that is produced after the application of heat and filtration to separate and remove the milk solids and water from yak butter or cream. Ghee has become increasingly popular in cuisines in India, China, and some African countries due to its culinary properties being closer to vegetable oil compared to butter. For instance, cow ghee has a higher smoke point (245 °C) than cow butter (191 °C) does, making it more convenient for frying foods [1]. Apart from frying, yak ghee is also used for sautéing, grilling, and baking at high temperatures. However, heating edible oils at temperatures of 170–200 °C induces a series of oxidation, isomerization, and polymerization reactions [2]. Oxidation reactions are also prevalent during prolonged or improper storage conditions and are a major cause of fat and oil deterioration. Oxidation is a three-phased reaction mechanism that involves free radical chain propagation reactions (also known as auto-oxidation) and the formation of primary and secondary oxidation products. These reactions do not only reduce the shelf-life, sensory, and nutritive quality of foods that have been heated/cooked with oil, but they also cause health problems such as cancers, cardiovascular diseases, and diarrhea when these foods are consumed [2,3,4]. Although oxidation is irreversible and inevitable, studies have shown that it can be delayed and its deteriorated products kept at acceptable levels with the addition of natural and artificial antioxidants [5,6]. In that regard, Kapadiya et al. [7] found that betel and curry leaves inhibited the oxidation of ghee during storage. The authors reported peroxide values of 4.99 meqO_2_/kg and 7.99 meqO_2_/kg in betel-flavored ghee and raw ghee, respectively, after accelerated storage studies (80 °C for 12 days). Another study discovered that butylated hydroxy anisol (BHA)-fortified ghee had the highest radical scavenging activity (91.77%) during accelerated storage and that ghee that had been fortified with steam-distilled clove extract had the highest oxidative stability during deep-frying [8]. 

There has been a paradigm shift in modern consumerism to less or non-chemically treated foods [9]. This increasing trend means that the use of artificial antioxidants such as BHA is not available for enhancing the oxidative stability of edible oils. In addition, high levels of butylated hydroxytoluene (BHT) and BHA have been linked to toxicity, including carcinogenesis [3]. This has motivated various research studies looking into the use of natural antioxidants to enhance the oxidative stability of edible oil. Furthermore, the incorporation of natural antioxidants into oils is not complete if the methods that are used in extracting these bioactive compounds do not use green solvents. It has been reported that the conventional organic solvent extraction methods, such as soxhlet extraction, leave hexane/petroleum-based solvent residues in the extract, resulting in toxicity and adverse health effects [10]. In view of this, many organic solvent-free extraction methods have been developed to produce safer products. These methods include the use of green solvents as extraction solvents, ultrasound-assisted extraction (UAE), and high shear dispersion (HSD) techniques. Baria et al. [10] and Tiwari et al. [11] reported that the HSD technique produced the highest carotenoid extraction (158.88% and 94.8%, respectively) when flaxseed was used as a green solvent. Corbu et al. [12] observed significantly higher carotenoid extraction using UAE than when using the maceration technique in olive oil. However, from literature, these modern methods have rarely been applied to ghee. 

*Lycium barbarum* L. (part of the Solanaceae family) is a 1–2 cm long reddish-orange berry with a sweet and tangy taste. Due to its richness in carotenoids, it has been added to olive and soya bean oils as an antioxidant to enhance the oxidative stability of those oils. This antioxidant ability has been associated with its zeaxanthin component [3,13,14]. Zeaxanthin is the major carotenoid in goji berry (over 80% of its total carotenoid content), followed by b-Cryptoxanthin [3,15], and in dairy, zeaxanthin is the carotenoid with the lowest concentration [16]. β-carotene is the main carotenoid in dairy, and is present in a range between 16.7 and 186.5 µg/100 g in milk and full-fat cream in Finland [16]. This study hypothesized that enriching yak ghee with goji berry carotenoids using a green solvent extraction method may produce a new type of yak ghee that is free of organic solvents and that has an enhanced oxidative stability during storage and heating. Recently, microwave oven heating (MW-heating) has become popular in food processing and in homes. This is due its green heating feature, which allows users to enjoy benefits such as industrial adaptability; flexibility of operation; low cost, energy consumption, and cooking time; and high maintenance of bioactive compounds. Microwave heating (MW-heating) is based on the conversion of electromagnetic energy into thermal energy by inducing the rotation of the dipoles in foods, and the heat is instantaneously produced by friction between molecules [17,18]. Currently, there is scarce information on the effect of MW-heating on the oxidative stability and fatty acids in ghee.

According to Peña-Serna et al. [19], cow and buffalo ghees have low nutritive values to humans due to their low levels of conjugated linoleic acid (CLA). However, it is anticipated that yak ghee would have an appreciable CLA content even after extensive storage and heating due to its high content in yak butter [20]. Therefore, the aim of this study is to enrich yak ghee with goji berry carotenoids by means of a green solvent extraction method and to determine the changes in color, total carotenoid content, free fatty acids, oxidative stability, radical scavenging activities, and fatty acid profiles of both types of yak ghee during MW-heating and accelerated storage. This study will provide vital information for dairy processors and marketers.

## 2. Materials and Methods

### 2.1. Preparation of Yak Ghee

Fresh yak butter was obtained from Qinghai Golden Qilian Dairy Co. Ltd. (Qindao, China), and the organic dried Ningxia goji berries (Ningji No.1 variety) were obtained from Ningxia Pure Goji Biology Technology Co. Ltd. and were milled. Yak butter was melted completely in a preheated oven at 50 °C to achieve prestratification, and the middle layer was filtered and divided into two parts. One part butter oil was further heated at 110 °C to clarify it, and it was then filtered with a cheese cloth that had been folded four to six times to obtain fresh yak butter ghee (FG0), which was set aside at −80 °C for further analyses and treatment. 

The second part was mixed with 5% goji berry powder, and the carotenoids were extracted [11,12]. The 5% goji berry enrichment was selected based on preliminary trials that showed that higher percentages of goji berry overshadowed the natural flavors of yak ghee (had lower taste and color acceptability values) and had higher peroxide values (pro-oxidation effect). The mixture was subjected to high shear dispersion for 10 min at 12,000 rpm and ultrasound-assisted extraction for 20 min using an XHF-D high speed dispensator, Ningbo Scientz Biotechnology, China, and a KQ-250 ultrasonic bath, Kunshan Ultrasonic Instrument Co. Ltd., China, (made of a 300 × 240 × 150 mm inner groove, an ultrasound power of 360 W, ultrasonic frequency of 40 KHz, and heating power of 600 W), respectively. The temperature of the ghee was maintained at 25 °C using cold water during its preparation. The mixture was allowed to settle, was filtered using a cheese cloth that had been folded four to six times and labeled as GG0. Both FG0 and GG0 were analyzed for their yield, moisture, solids-not-fat (SNF), and fat content and were divided into storage and heated samples.

### 2.2. Yield, Moisture, Solids-Not Fat (SNF), and Fat Content of Yak Ghee

The ghee yield% was calculated as the percentage ghee per 100 g of yak butter. 

The moisture content% of the ghee was determined by the oven drying method using the net weight of the ghee after drying the sample to a constant weight at 102 ± 2 °C. 

The solids-not-fat (SNF%) was determined by weighing 10 g of each ghee sample in a metal dish. Water was evaporated as described in the moisture content analyses. Furthermore, 30 mL of petroleum ether was added, and the dish was placed in the oven at 102 ± 2 °C for 1 h, after which it was removed and cooled in a desiccator for 30 min. Sixty (60) mL of petroleum ether was added to each dish, and the contents were mixed thoroughly. The dish was positioned in a sloping position to allow the SNF to settle, and the solvent was decanted. The fat extraction step was repeated using 60 mL of solvent. The dish was then heated at 102 ± 2 °C in the oven until all of the solvent and moisture had been removed and the SNF was thoroughly dried. Fat extraction was repeated with 40 mL and 20 mL petroleum ether. In between the two extractions, the SNF was dried to a constant weight, cooled in the desiccator for 30 min [21], and calculated as follows:

(1) $$\mathrm{SNF}\%=\frac{\left(\mathrm{final}\;\mathrm{weight}-\mathrm{empty}\;\mathrm{dish}\;\mathrm{weight}\right)\times 100}{\mathrm{initial}\;\mathrm{weight}\;\mathrm{of}\;\mathrm{ghee}}\;$$

The Fat content% was evaluated by weighing five grams of each ghee sample in a centrifuge tube. Then, 20 mL of petroleum ether was added and mixed thoroughly with the vortex mixer. The mixture was centrifuged at 100× *g* for 5 min. The solvent phase was transferred to a collecting vessel (pre-dried and pre-weighed metal dish). The extraction step was repeated twice on the non-soluble part in the centrifuge tube with 10 mL petroleum ether, and the solvent phase was transferred to the collection vessel. The mixture was dried at 102 ± 2 °C for 30 min to remove all of the solvent. The drying step was repeated until the difference between two consecutive weights did not exceed 1.0 mg or until the mass increased [21]

(2) $$\mathrm{Fat}\%=\frac{\left(\mathrm{final}\;\mathrm{net}\;\mathrm{weight}\;\mathrm{of}\;\mathrm{ghee}\;\right)\times 100}{\mathrm{initial}\;\mathrm{weight}\;\mathrm{of}\;\mathrm{ghee}}$$

### 2.3. Microwave Heating (MW) and Accelerated Storage

Microwave heating (MW-heating) analyses were carried out using a commercial microwave oven from the Appliance Company of America, ACA model TM33HT, with 1700 W and 230 °C as the maximum wattage and temperature. An amount of 200 g of each ghee sample was placed in non-stick carbon steel rectangular baking tray (20 cm × 15 cm × 1 cm) and exposed to preselected internal MW temperatures of 180 °C (15 and 30 min) and 200 °C for 30 min. These heating conditions were selected since frying and baking (common culinary uses of ghee) are commonly conducted at those conditions. The MW energy delivered to each sample was calculated as E = Wtm^−1^: where W is the microwave oven wattage, t is the time (min) of MW exposure, and m equals sample quantity, resulting in 99.75 KJg^−1^, 199.56 KJg^−1^, and 221.74 KJg^−1^, respectively (assumption: the wattage of each temperature selected was a proportion of 1700 W). The heated oils were collected, filtered, and stored at −80 °C until further analyses. The FG0 and GG0 samples were poured into brown bottles, corked, stored in the oven for 30 days at 65 °C, and analyzed after every 10 days. The samples were evaluated for peroxide value, acid value, Thiobarbituric acid (TBA), 2,2′-Azinobis(3-ethylbenzothiazoline-6-sulfonic acid) (ABTS) radical scavenging activity, 2,2-diphenyl-1-picrylhydrazyl (DPPH) radical scavenging activity, fatty acids, color, and total carotenoids [17,22].

### 2.4. Color Assessment

The color values of 20 g of each ghee sample that had been placed in a Petri dish was measured at 25 °C using the Konika Minnolta’s Chroma meter CR-140 (Tokyo, Japan) to obtain the L*a*b* values of each sample. The equipment was calibrated using the manufacturer’s white and black board. Using the CIELAB color system, where the psychometric index of L* refers to lightness (ranging from black = 0 to white = 100); a*, indicates the red/green color coordinates of the ghee, and b* refers to the yellow/blue parameters, triplicates of each sample were recorded. To evaluate the color differences (ΔE^*^_ab_) between FG0 and GG0 ghee samples after MW-heating and accelerated storage, the Euclidean distance between the two colors in the three-dimensional L*a*b* color space was calculated using the formula

ΔE^*^ _ab_ = √ (ΔL*)² + (Δa*)² + (Δb*)²; (3)

where ΔL*, Δa*, and Δb* are differences between FG0 and GG0 during storage and heat treatment.

Chroma values were calculated using C = √ (a*^2^ + b*^2^), where a* and b* are the color values of the samples [17].

### 2.5. Total Carotenoid Content (TCC)

An amount of 1 g of goji berry powder or 1 mL yak ghee sample mixed with 5 mL acetone (75%) was allowed to stand for 30 min, and 2.5 mL of hexane was added to the mixture, which was then stirred for 30 min. In addition, 15 mL of hexane: acetone: ethanol in the ratio 2:1:1 was added and agitated for 30 min followed by the addition of 2.5 mL distilled water, and the mixture then agitated again for 5 min. The mixture was centrifuged at 3000 rpm for 10 min, and the upper layer was removed. Another extraction was conducted with 15 mL hexane, and the total volume was carefully set aside for TCC calculation. Absorbance was read at 450 nm using hexane as the blank. TCC was calculated using the formula

(4) $$\mathrm{TCC}=\frac{A\times V\times 1000}{P\times \varepsilon }$$

where A is the absorbance at 450 nm, V is the total volume of the hexane layer (mL), ε (A^1%^ _1cm_) is the extinction co-efficient of the carotenoid mixture in hexane (2500 dL/g cm), and P is the weight (g) or volume (mL) of the sample [10].

### 2.6. Acidity Value (AV) 

The acid value of the yak ghee was evaluated using the procedure described by Piotr et al. [23]. An amount of 10 g of butter was dissolved in 50 mL of a petroleum ether: alcohol (2:1) mixture and was titrated against 0.1 M standardized alcoholic KOH using phenolphthalein as the indicator. The acid value (AV) is the KOH in mg used to neutralize 1.0 g of butter and was calculated with the formula

(5) $$\mathrm{AV}\;\left(\mathrm{mg}\;\mathrm{KOH}/g\right)=\frac{56.11\times 0.1\times \mathrm{titer}}{\mathrm{mass}\;\mathrm{of}\;\;\mathrm{butter}}$$

Titre values were blank-corrected without ghee.

### 2.7. Peroxide Value (PV) 

To determine the peroxide concentration, the method byAdjonu et al. [24] was used. An amount of 2 g yak ghee was melted at 30 °C and dissolved in 30 mL trichloromethane to create a glacial acetic acid (2:3) mixture. Subsequently, 1 mL of saturated KI solution was added, and the mixture was shaken for 60 s. The mixture was then placed in the dark for 5 min, after which 100 mL distilled water was immediately added. The mixture was titrated with 0.01 N Na_2_S_2_O_3_ to a yellow color, and exactly 1 mL of a 1% starch solution was added (color changed to blue), and titration continued until the deep blue color disappeared.

(6) $$\mathrm{PV}\;\left(\mathrm{meq}.\;O2/\mathrm{kg}\;\mathrm{of}\;\mathrm{butter}\right)=\frac{S\times N\times 1000}{g}$$

where S = mL Na_2_S_2_O_3_ (blank corrected) and N = normality Na_2_S_2_O_3_ solution, and g = mass of the ghee.

### 2.8. Thiobarbituric Acid (TBA) Test 

The thiobarbituric acid value of the yak ghee was evaluated using Chinese National Standard (CNS), GB_T 35252-2017 [25]. An amount of 10 g yak ghee was melted at 30 °C with 1 g anhydrous sodium sulfate, and the clarified oil was filtered. TBA reagent was prepared by diluting 200 mg TBA in 100 mL n-butanol, which was then for 12 h at room temperature and then filtered. Then, 200 mg of ghee and 25 mL n-butanol were mixed thoroughly. Furthermore, 5 mL each of the sample solutions and TBA reagent were pipetted into centrifuge tubes and heated in a water bath at 95 °C for 2 h. Samples were cooled to room temperature; absorbance was measured at 530 nm using a visible spectrophotometer (Shanghai Spectral Instrument Company Limited, model 723, Shanghai, China). The results were calculated using the formula below:

(7) $$\mathrm{TBA}\;\left(\mathrm{mgMDA}/\mathrm{kg}\right)=\frac{50\left(A-B\right)}{m}$$

where A = sample absorbance, B = blank absorbance, and m = mass

### 2.9. 2,2-Diphenyl-1-Picrylhydrazyl (DPPH) Radical Scavenging Activity 

To determine the radical-scavenging activity (RSA) of yak ghee, 0.5 mL yak ghee was dissolved in 0.5 mL ethyl acetate and vortexed for 30 sec. Subsequently, 0.15 mL yak ghee solution was then mixed with 3.350 mL of DPPH solution (0.04 mM in ethyl acetate) in a centrifuge tube. The mixture was vortexed for 10 s, incubated for 30 min in the dark, and the absorbance was measured at 517 nm using a visible spectrophotometer (Shanghai Spectral Instrument Company Limited, model 723, Shanghai, China). The absorbance of the ethyl acetate DPPH solution was used as the reference (control). The RSA of the yak ghee sample was calculated by the percentage of DPPH scavenging activity [26].

(8) $$\mathrm{DPPH}\%=1-\frac{\mathrm{Sample}\;\mathrm{absorbance}\;}{\mathrm{Control}\;\mathrm{absorbance}}\times 100\%$$

### 2.10. 2,2′-Azinobis(3-Ethylbenzothiazoline-6-Sulfonic Acid) (ABTS) Radical Scavenging Activity 

To determine the RSA by means of the ABTS assay, an ABTS radical stock solution was first prepared by reacting equal volumes of 7.00 mM ABTS diammonium salt and 2.45 mM potassium persulfate solution in the dark for 13 hrs. Then, the solution was diluted with 95% ethanol to attain an absorbance of 1.0 nm. To every 200 µL yak ghee solution (0.01 g ghee dissolved in 10 mL 1-butanol), 6.00 mL ABTS solution was added, and absorbance at 734 nm was measured after 15 min using a visible spectrophotometer (Shanghai Spectral Instrument Company Limited, model 723, Shanghai, China). Reference control was determined with a mixture of 6.00 mL ABTS solution and 200 µL 1-butanol. The RSA of yak ghee was calculated as follows [26]:

(9) $$\mathrm{ABTS}\%=1-\frac{\mathrm{Sample}\;\mathrm{absorbance}\;}{\mathrm{Control}\;\mathrm{absorbance}}\times 100\%$$

### 2.11. Fatty Acids Analyses 

#### 2.11.1. FAME Preparation

For the pretreatment of the ghee samples and the preparation of fatty acid methyl esters (FAME), the procedure by Bligh and Dyer [27] was followed with slight modifications. An amount of 2 g of ghee was dissolved in a mixture of 112.5 mL chloroform and 37.5 mL methanol, homogenized, and filtered, and 25 mL of a saturated NaCl solution was added. The lower organic phase was separated after standing for 5 min and was dehydrated with anhydrous sodium sulfate, and the solution was then again allowed to stand before it was filtered with sodium sulfate and washed with the chloroform–methanol solution (3:1). The fat mixture was concentrated with rotary evaporation at 40 °C, and 100 mL of 2 mol/L NaOH–methanol was added to a water bath at 50 °C and heated for 15 min. The mixture was transferred into a separating funnel, 100 mL of mol/L HCl and 50 mL of ether were added, and the mixture was subsequently allowed to stand, and the upper organic solvent was recovered. A 5 mL boron trifluoride–methanol was added, and the mixture was heated a 60 °C in a water bath for 1 h, and extract was recovered with 10 mL of ether using a separating funnel. The extract was remixed with 10 mL of distilled water and 20 mL ether, and the upper layer was recovered and concentrated for GC-MS analysis.

#### 2.11.2. GC-MS Parameters

Gas Chromatographic Conditions: Gas chromatograph 6890 N; elastic quartz capillary column was SE-54, 30 m × 0.25 mm × 0.25 μm; gasification chamber temperature 250 °C; temperature program: 80–290 °C; temperature program: kept at 80 °C for 1 min, increased to 280 °C at 8 mL/min, kept for 30 min; carrier gas: He; carrier gas flow: 1.2 mL/min; pressure 2.4 kPa; linear velocity: 40 cm/s; volume delay: 3 min; injection volume: 1 μL. Split ratio: 50:1. 

Mass Spectrometry Conditions: Mass spectrometer was 5973 N quadrupole mass spectrometer; ion source: electron bombardment ion source; ion source temperature: 230 °C; transmission line temperature 150 °C; ionization energy of ion source: 70 ev; mass scanning range m/z: 33–660. 

Data Analysis: Each component was analyzed by GC-MS, and the mass spectrum corresponding to each peak was compared to the NIST database to determine the compound type and the relative content of each fatty acid component in the ghee sample.

### 2.12. Statistical Analysis

All experiments were repeated in triplicate and analyzed using Excel 2016. Data from the sample were presented as mean and SD and analyzed via one-way analysis of variance (ANOVA) and Tukey’s post hoc test using SPSS (Statistical Package for the Social Sciences 22.0) software (Chicago, IL, USA). *p* < 0.05 was considered statistically significant in the sample groups for all of the analyses. The two-sample *t*-test by GenStat 12.1 (VSN international Ltd., Hempstead, United Kingdom) was used to determine significant differences between the fatty acids of the two types of yak ghee.

## 3. Results and Discussion

### 3.1. Physical Properties of Yak Ghee Samples

Incorporating *Lycium barbarum* L. carotenoids into yak ghee (GG0) by high shear dispersion (HSD) and ultrasound-assisted extraction (UAE) resulted in lower moisture and fat contents but higher yield and SNF. However, there was no SNF in raw the yak ghee (FG0) samples due to the various separation steps involved in its preparation (Table 1). The agitation steps involved in HSD and UAE probably enhanced the moisture evaporation from GG0, resulting in lower moisture.

Moisture is very important in ghee preparation, as high values enhance lipid oxidation and lowers the shelf life of ghee [28]. According to the Codex Standard, ghee contains 99.60% fat, no SNF, and no moisture [29]. However, previous studies have shown that ghee from other ruminants such as buffalo and cow milk comprise a moisture to fat ratio of 0.09–0.50%:96.00–99.50% [28,30,31,32]. Thus, the moisture and fat contents of FG0 and GG0 agreed with previous studies. Cheese cloth sieving did not effectively remove all of the SNF from GG0; therefore, centrifugation should be used in subsequent studies.

### 3.2. Color Assessment

**Figure 1:**
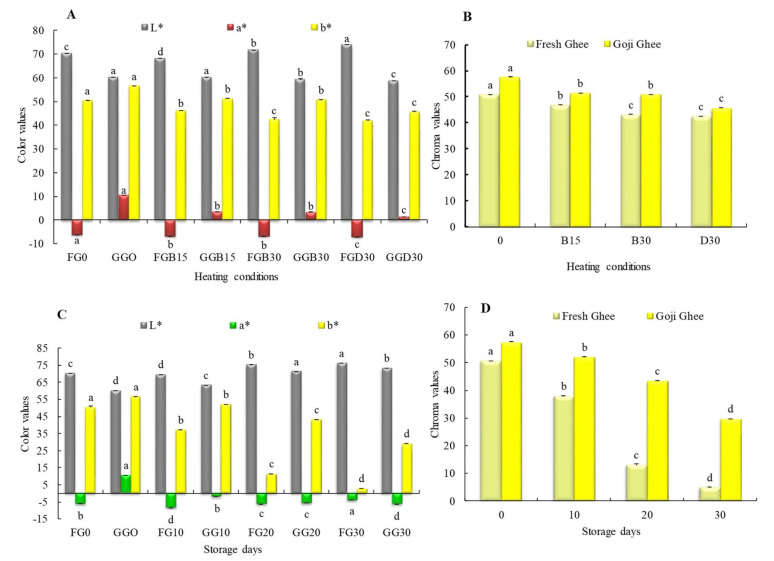
Color values of FG0 and GG0 (**A**). Changes in L*a*b* of FG0 and GG0 during MW-heating; (**B**). Chroma values of FG0 and GG0 during MW-heating; (**C**). changes in L*a*b* of FG0 and GG0 during accelerated storage; (**D**). Chroma values of FG0 and GG0 during accelerated storage (Figure 1B,C). The labels 0, B15, B30, and D30 represent color values at zero and the MW temperatures 180 °C (15 and 30 min) and 200 °C for 30 min, respectively. Error bars represent the standard deviation (sd) of the mean value (*n* = 3). “a, b, c, d” indicate significant differences within individual ghee treatments at *p* < 0.05.

Ghee color (as recorded in terms of CIE L*a*b* color scale) is influenced by factors such as dairy source, processing method, the part of the ghee analyzed (outside or inside), and the ghee composition [28,33]. In this study, during MW-heating, the lightness (L*) of the FGO samples increased from 70.16 to 73.96 (indicating a paler color), while that of the GGO samples decreased from 60.05 to 58.72 (indicating a darker color (Figure 1A). The accelerated storage of the FG0 and GG0 resulted in a significant increase in the whiteness from 70.16 to 76.14 and from 60.05 to 73.22, respectively (Figure 1C). Suwarat and Tungjaroenchai [28] observed a whiter color in the fresh buffalo ghee (L*: 100.95 to 94.91) than that observed in the FG0 and GG0 at zero due to the lack of carotenoids in buffalo milk [28]. The a* parameter geared towards a green color decreased from −6.23 to −7.11 and from 10.39 to 1.35 in the FG0 and GG0 samples, respectively, during MW-heating, though GG0 was still redder than FG0, as expected. From the initial values of the a* parameter, all of samples from both types of ghee became greener during accelerated storage. The FGO, which was already in the green region, increased to −3.97, while the GGO decreased from being initially in the red region to being in the green region (−6.32) as the storage duration increased *(p* < 0.05). The yellow color (b*) was deeper in the GG0 samples than it was in the FGO samples due to the enrichment from the goji berry carotenoids; values ranged from 56.33 to 45.50 and from 50.24 to 41.81, respectively, during MW-heating. Therefore, there was significant decrease in the yellow color in both types of ghee as the MW-heating temperature and duration increased *(p* < 0.05). Under accelerated storage, the yellowness (b*) of both types of ghee showed a decreasing trend, with FG0 losing almost all of its yellowness (2.74 at 30 days), and GG0 showed a decreasing trend to 28.98 after 30 days of storage (Figure 1A,C).

The increase in whiteness and loss in yellowness observed in FG0 during MW-heating and storage and GGO during storage was due to carotenoid degradation [34]. The GGO darkening that took place during MW-heating could be attributed to the accumulation of non-volatile decomposition products [35], the burning of suspended carotenoids, or Maillard browning (MB). Maillard browning is a series of complex reactions that takes place between carbonyls (such as starches, and sugars) and amino acids (such as methionine) that result in the darkening and burnt flavors of in foods [36]. These carbonyls and amino acids were probably introduced into the yak ghee from the goji berry powder. A previous study showed that MW-heating advanced the production of MB intermediates such as 4,5-dimethyloxazole and 2,3-dihydro-3,5-dihydroxy-6-methyl-4Hpyran-4-one [37]. Other authors observed that the b* parameter was directly correlated with carotenoids such as trans-βcarotene, vitamin A, and other pigments in milkfat [33,38]. Therefore, enriching yak ghee with goji berry carotenoids curtailed the complete loss of yellow color in yak ghee during storage. Chroma values showing the color intensity of yak ghee was higher in the GG0 than samples than it was in the FG0 samples and was significantly decreased during MW-heating and storage, as shown in Figure 1B,D. However, it was observed that there was no significant difference between the chroma values of FGB30 and FGD30 as well as in the chroma values of GGB15 and GGB30 (*p* < 0.05). The color differences (ΔE^*^_ab_) in the MW-heated samples were 20.39 ± 0.57, 15.96 ± 0.34, 18.07 ± 0.72, and 17.44 ± 0.21. However, the ΔE^*^_ab_ in the yak ghee samples under accelerated storage were 20.39 ± 0.57, 17.52 ± 0.26, 31.92 ± 0.85, and 26.47 ± 0.18 (from 0 to 30 days of storage). These results show that there was a clear distinction in the color differences between the FG0 and GG0 samples and that these differences could be easily perceived with the human eye since in dairy samples, only ΔE^*^_ab_ < 3 cannot be perceived [33]. The ability of the goji berry carotenoids to maintain the yak ghee color over a long period of time is advantageous to ghee producers and marketers.

### 3.3. Total Carotenoid Content

**Figure 2:**
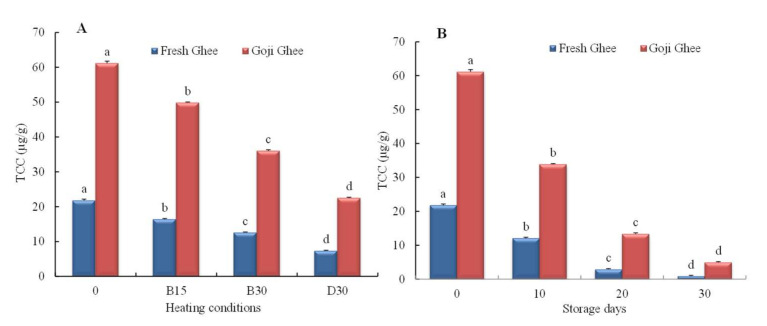
(**A**). Total carotenoid content of yak ghees during MW-heating; (**B**). Total carotenoid content of yak ghees during accelerated storage. Error bars represent the standard deviation (sd) of the mean value (*n* = 3). The labels 0, B15, B30 and D30 represent AV, PV, and TBA values at zero and the MW temperatures 180 °C (15 and 30 min) and 200 °C for 30 min, respectively. “a, b, c, d” indicate significant differences within individual ghee treatments at *p* < 0.05.

In this study, the total carotenoid content (TCC) of goji berry was 3778.67 ± 8.33 µg/g, while that of FG0 and GGO were 21.78 ± 0.39 and 61.17 ± 0.60 µg/g; hence a 180.85% increment in the TCC of yak ghee was achieved. The combination of UAE and HSD used in this study extracted higher goji berry carotenoid concentrations when compared to the results reported by Baria et al. [10]. The authors achieved a maximum carotenoid extraction of 158.88% into flaxseed oil after using 20,000 rpm HSD for 4 min or 8 min and had the poorest extraction when they used UAE from 2 min to 8 min. HSD uses high shear action between its static and rotating probe to disrupt the stiff food matrix that releases carotenoids. However, UAE uses acoustic cavitation to break down the cell walls to free the carotenoids and pigments embedded in the cell [10,39]. After heating at 200 °C for 30 min, MW-heating decreased the TCC of FG0 and GG0 by 67.53% and 63.14%, respectively, with the remaining TCC of GG0 being 67.55% higher than that of FG0. This signifies that MW-heating had a higher degrading effect on FG0 than it did on GGO. The storage of FG0 and GG0 at 65 °C showed a significant decrease in TCC every 10 days, until final concentrations of 0.87 ± 0.23 µg/g and 4.96 ± 0.19 µg/g (99.13% and 91.89% decrease), respectively, were reached, as presented in Figure 2 (*p* < 0.05). This signifies the protective effect of the goji berry carotenoids on yak ghee during MW-heating and storage.

**Figure 3:**
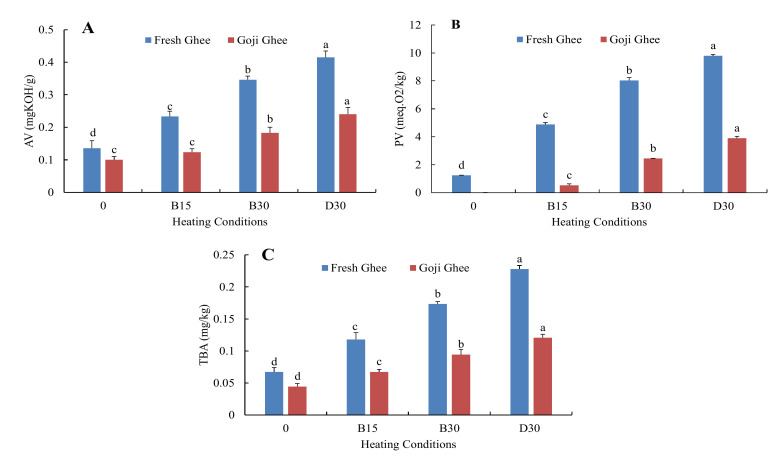
Changes in free fatty acids and oxidative stability of FG0 and GG0 during MW-heating. (**A**). Acid values. (**B**). Peroxide values. (**C**). Thiobarbituric acid values. The labels 0, B15, B30, and D30 represent AV, PV, and TBA values at zero and the MW temperatures 180 °C (15 and 30 min) and 200 °C for 30 min, respectively. Error bars represent the standard deviation (sd) of the mean value (*n* = 3). “a, b, c, d” indicate significant differences within individual ghee treatments at *p* < 0.05.

In this study, the severe TCC loss that occurred during the prolonged exposure of both types of yak ghee to MW-heating and accelerated storage could be attributed to a loss in β-carotene since there is a positive correlation with the b* color parameter and TCC (β-carotene is responsible for the yellowness of ghee). Therefore, a significant loss in its content would result in a significant decrease in the TCC of yak ghee. Being unsaturated, β-carotene is highly reactive with the molecular oxygen present in yak ghee, which makes it susceptible to many degradation and oxidative reactions [40]. The high content of β-carotene in milk fat along with β-cryptoxanthin is very important to human health due to its critical role in pro-vitamin A activity and antioxidant activity. Meanwhile, zeaxanthin together with other carotenoids such as lutein is also involved in antioxidant activities [17]. A similar TCC loss trend was observed when the edible oils were heated for 1–15 min in a 1000 W microwave oven [41,42]. Aside from β-carotene, the TCC loss in yak ghee during extensive microwave heating and storage could be linked to losses in other carotenoids through isomerization (trans- carotenoids to cis-carotenoids) as well as oxidation. Although minimal MW-heating such as pasteurization (for 1 to 3 min) does not cause significant changes in carotenoids in foods, higher MW-heating induces the disappearance and isomerization of some carotenoids into other forms, signifying the loss of function [18,41]. For instance, as MW-heating leads to thermal oxidation, increasing the acid value of ghee, as shown in AV results in Figure 3A, would result in the 5,6-epoxycarotenoids present isomerizing to 5,8-epoxyfuranoids [43].

Furthermore, a high storage temperature coupled with a prolonged storage time enhances epoxidation, the formation of apocaroteniods, and hydroxylation. These reactions result in the loss of carotenoid colors and biological function as well as the synthesis of low molecular and volatile compounds that give off-flavors [44,45]. Decreases in the carotenoids during storage have been reported in other edible oils. Montesano et al. [14] observed a slight decrease in the zeaxanthin content of olive oil when goji berry carotenoids were added during storage at 20 °C for 28 days. Contrary to this study, Blasi et al. [3] reported lower levels of carotenoids such as lutein and zeaxanthin dipalmitate in olive oil when 1.5 mg/100 g oil of organic solvent-extracted goji berry carotenoids was added. The authors also observed a slightly higher decrease in the carotenoid content in fortified olive oil than in the control when both oils were open air-fried at 180 °C for 20 min. These differences could be due to differences in extraction method and in the concentrations of the goji berry carotenoids. Therefore, enriching yak ghee with goji berry carotenoids by UAE and HSD does not only improve the carotenoid content in yak ghee but also maintains it during processing and storage. 

### 3.4. Free Fatty Acids (FFA) and Oxidative Stability (AV, PV and TBA)

The acid value (AV) is a critical indicator of the free fatty acid content resulting from the hydrolytic breakdown of the triglycerides (TAGs) in the ghee and indicates the extent of deterioration (rancidity) resulting from poor storage or processing conditions [46]. In lipid hydrolysis, lipases catalytically cleave off the three fatty acids from the glycerol backbone of unsaturated fatty acids (UFA) during the second stage of lipid oxidation. High levels of these FFA, especially short-chain free fatty acids, result in the butter demonstrating rancidity and off-flavors that are unacceptable to consumers [47]. However, the peroxide value (PV) measures the concentration of primary oxidation products (peroxides) that serve as an integral marker of lipid oxidation in ghee. At the initial stages of lipid oxidation, peroxides are produced from UFA reactions with molecular oxygen [48]. Furthermore, TBA (TBARs) measures malondialdehyde (MDA) mg as well as other reactive substances such as 2-alkenals and 2,4-alkadienals in 1 kg of a substance. MDA (α,β-unsaturated aldehydes) is the most abundant aldehyde produced together with ketones, epoxides, hydroxy compounds, oligomers, and polymers from the oxidation of the lipid peroxides formed from primary oxidation when exposed to oxygen. These secondary oxidation products are what give oxidized products their distinctive off-flavors [46,49].

**Figure 4:**
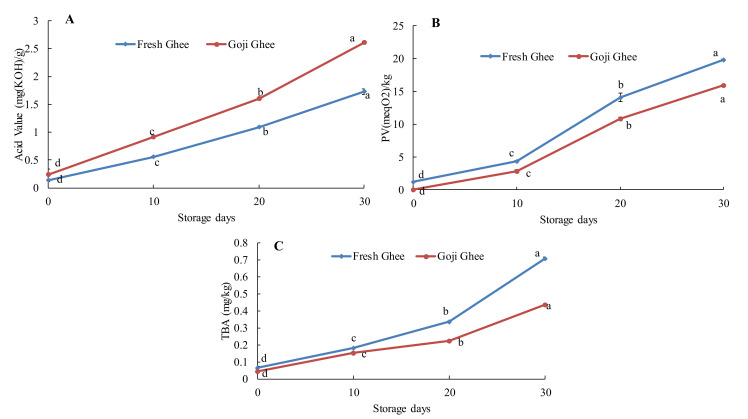
Changes in the free fatty acids and oxidative stability of FG0 and GG0 during accelerated storage. (**A**). Acid value. (**B**). Peroxide value. (**C**). Thiobarbituric acid values. Error bars represent the standard deviation (sd) of the mean value (*n* = 3). “a, b, c, d” indicate significant differences within individual ghee treatments at *p* < 0.05.

In this study, the AV, PV, and TBA of FG0 were 0.14 mgKOH/g, 1.24 meqO_2_/kg, and 0.07 mg/kg, respectively (Figure 3). While there were no peroxides in GG0, the initial AV and TBA values were 0.1 mgKOH/g and 0.04 mg/kg, respectively. In addition, MW-heating significantly increased these parameters after each heating condition, as presented in Figure 3 (*p* < 0.05). The AV, PV, and TBA of FG0 increased to maximum values of 0.41 mgKOH/g, 9.80 meqO_2_/kg, and 0.23 mg/kg, respectively. While the AV, PV, and TBA of GG0 increased to 0.24 mgKOH/g, 3.89 meqO_2_/kg, and 0.12 mg/kg, respectively. As presented in Figure 4, accelerated storage showed a similar but increased trend to that of MW-heating in FFA and in the oxidative stability parameters. The AV, PV, and TBA of FG0 increased to 1.73 mgKOH/g, 19.84 meqO_2_/kg, and 0.71 mg/kg, respectively, after 30 days of storage. While the AV, PV, and TBA of GG0 increased to 0.88 mg KOH/g, 15.94 meqO_2_/kg, and 0.44 mg/kg, respectively, after 30 days of storage. One-way ANOVA showed that these increments were statistically significant for every 10 days of storage (*p* < 0.05). These results indicate that incorporation of goji berry carotenoids into yak ghee produced more stable yak ghee than the control. Furthermore, MW-heating and accelerated storage had a more degrading effect on the oxidative stability of FGO than it did on that of GG0. Ultimately, this study showed that enriching yak ghee with goji berry carotenoids inhibited the production of peroxides, MDA, and free fatty acids. This effect is mainly due to increased levels of the carotenoid antioxidants such as zeaxanthin that the goji berries probably enriched in GG0.

Corbu et al. [12] reported significantly lower PV in edible oil with 10% carotenoid enrichment by UAE than in oil samples with a lesser percentage of enrichment. In addition, the higher rate of thermal oxidation in the unsaturated fatty acids (UFA) oinFG0 during MW-heating is a major factor, as explained by Dostálová et al. [50]. It has been reported that the oxidative stability of oils stored at 65 °C for 24 h is the same as storage for one month at room temperature [22]. This implies that, this study predicts the oxidative stability of yak ghee over the course of 2 and half years of storage and between the two types of ghee; GG0 had a longer shelf-life. The findings from this study were consistent with those by Kishorkumar and Aparnathi [7], who observed a range from 0 to 40.99 meqO_2_/kg in the PV of ghee stored under 80 °C for 288 h. Previous studies using conventional ghee frying/heating methods have reported similar increasing trends in AV, PV, and TBA with higher values. Zeb and Uddin [51] reported an increase in the FFA from 1.17 to 5.1% when desi cow ghee was heated at 160 °C for 9 h. The authors recorded 1.7 meqO_2_/kg PV in fresh ghee (control). Shende et al. [8] observed the absence of peroxides in fresh ghee and almost 12 meqO_2_/kg PV after open-air frying cotton balls for 28 min at 180 ± 5 °C in ghee. The authors also reported TBA values that were 16% to 30% higher compared to the values obtained in this study (FGB30-GGB30). The lower values reported in this study may be due to differences in heating temperatures, heating methods, ghee preparation methods, and the sources of ghee used.

### 3.5. Radical Scavenging Activity (RSA) 

**Figure 5:**
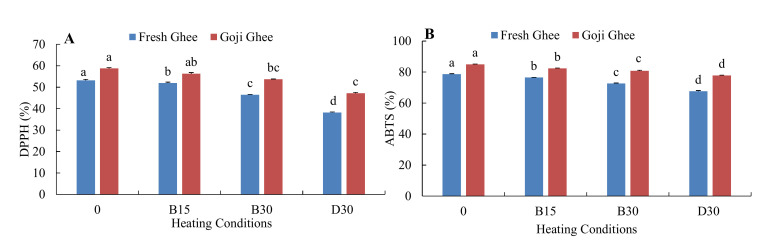
Changes in the radical scavenging activities of FG0 and GG0 during MW-heating. (**A**). DPPH values and (**B**). ABTS values. The labels 0, B15, B30, and D30 represent the DPPH and ABTS values at zero and the MW temperatures 180 °C (15 and 30 min) and 200 °C for 30 min, respectively. Error bars represent the standard deviation (sd) of the mean value (*n* = 3). Different lowercase letters in the same test indicate significant differences.

**Figure 6:**
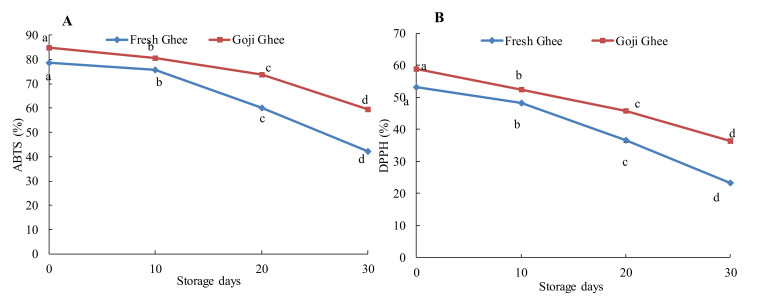
Changes in the radical scavenging activities of FG0 and GG0 during accelerated storage. (**A**). ABTS values; (**B**). DPPH values. Error bars represent the standard deviation (sd) of the mean value (*n* = 3). “a, b, c, d” indicate significant differences within individual ghee treatments at *p* < 0.05.

The RSA of the yak ghee samples in terms of the DPPH and ABTS values are depicted in Figure 5 and Figure 6. Increased MW-heating significantly decreased the DPPH values of FG0 and GG0 from 53.17–38.25% and from 58.77 to 47.20%, respectively, while the ABTS values of FG0 and GG0 decreased from 78.61 to 67.60% and 84.99 to 77.86%, respectively. Under accelerated storage, the DPPH values of FG0 and GG0 decreased to 23.19% and 36.38%, respectively, and the ABTS values decreased to 42.29% and 59.37%, respectively, after 30 days of storage (Figure 6). 

The decrease in the RSA of yak ghees was due to the decrease in the antioxidant compounds concentrations, as they were consumed by scavenging free radicals. Moreover, the higher MW-heating and storage temperature and duration increased the rate of free radical synthesis [52]. The depletion of yak ghee antioxidants occurs under numerous reduction-oxidation reactions that result in the oxidation of antioxidants. Carotenoids such as β-carotene, zeaxanthin, and lutein react with peroxide radicals to deactivate them (radicals) and to form resonance-unreactive carbon-centered radical adducts [53,54,55]. However, FG0 had a high antioxidant ability (RSA) when compared to initial the DPPH value of the control ghee by Asha et al. [53] (which was 30% on average). This may be due to yak dairy having higher amounts of antioxidant compounds than the levels in the dairy used by the researchers.

The antiradical actions of yak ghee are the combined effects of the antioxidant activities of all of the antioxidants in yak ghee, including carotenoids, alpha tocopherols, and enzymatic antioxidants. GG0 was enriched with goji berry carotenoids to boost the RSA of yak ghee during heating and storage, and from the results, its RSA was significantly enhanced compared to that of the control (*p* < 0.05). This observation is in agreement with changes in the AV, PV, and TBA values in both types of yak ghee. In a nutshell, the increased carotenoid content in GG0 inhibited oxidation (enhanced its oxidative stability), gave used yak ghee the potential to be reused, increased shelf life, and could prolong the duration of consumer acceptability. It is worth noting that this study did not show the pro-oxidation effect reported for carotenoid enrichments revealed in other studies [12,56]. The pro-oxidation effect of carotenoids is due to various ratios among the carotenoids and the other compounds involved [57]. 

### 3.6. Fatty Acids Analyses

**Figure 7:**
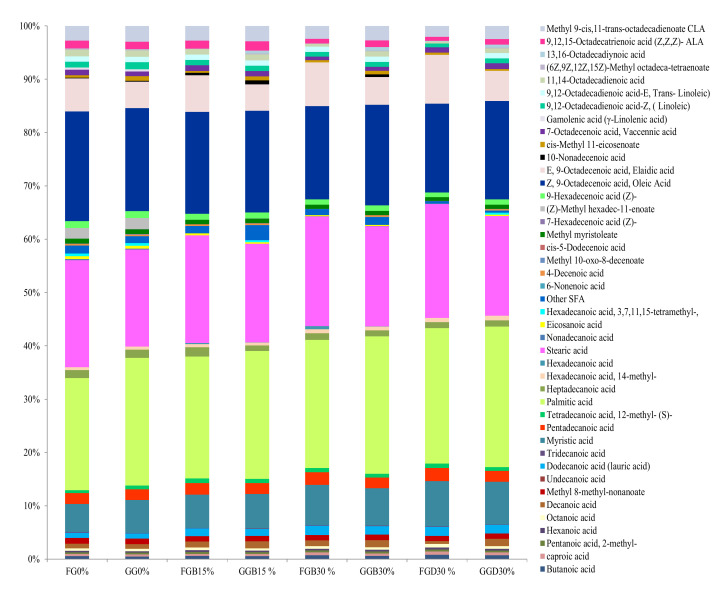
Changes in the fatty acid profiles of FG0 and GG0 during MW-heating. The labels 0, B15, B30, and D30 represent fatty acid values at zero and the MW temperatures 180 °C (15 and 30 min) and 200 °C for 30 min, respectively.

**Figure 8:**
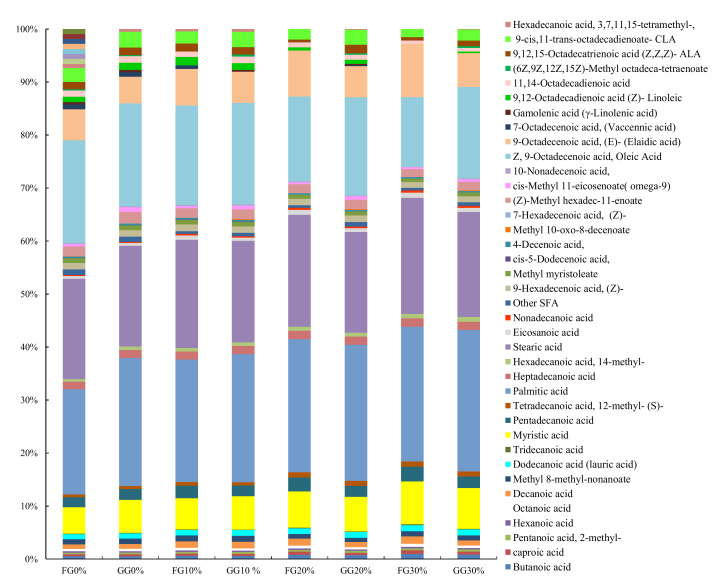
Changes in fatty acid profiles in FG0 and GG0 during accelerated storage. The labels 0, 10, 20, and 30 attached to FG and GG represent the storage days of raw yak ghee and goji berry carotenoid-enriched yak ghee.

The changes in the fatty acid profiles of FG0 and GG0 during MW-heating and accelerated storage are shown in Figure 7 and Figure 8. In agreement with previous studies [20,58], the SFA comprised the highest portion of fatty acids in both FG0 and GG0, containing palmitic, stearic, and myristic acids. It was observed that among these three SFA, the palmitic and myristic acids were significantly higher in GG0, and together with minor SFA, they increased the total SFA in GG0 by 3.50% (*p* < 0.05) (Table 2). However, as the temperature and duration of MW-heating progressed, with the exception of palmitic acid, most of the other SFA increased at a faster rate in FG0 to the extent of a 3% rise above the SFA of GG0 (FFD30% vs. GGD30%). Accelerated storage caused the SFA content in FG0 and GG0 to increase by 19.17% and 9.54%, respectively, after 30 days (Table 2). Palmitic, stearic, and myristic acids followed a similar pattern as the MW-heated yak ghee samples (Table 3). This study supports previous studies reporting significant increases in major SFA such as palmitic acid and stearic acids in edible oils during accelerated storage and MW-heating [22,59].

The MUFA content was 3.88% higher in FG0 than it was in GGO. At the highest MW-heating condition, FG0 lost 24.10% of its MUFA content, and GG0 only lost 9.94%. However, under accelerated storage, the MUFA of FG0 and GG0 decreased by 33.48% and 14.14%, respectively (Table 3). This study agrees with Blasi et al. [3], who reported a smaller MUFA loss in edible oil with added goji berry carotenoids after frying. MUFA loss can be attributed to a significant loss in oleic acids. After MW-heating at 200 °C, FF0 and GG0 showed 22.10% and 9.93% loss, respectively (Table 2). In addition, after 30 days of storage, the oleic acid in FG0 and GG0 reduced to 63.48% and 88.98%, respectively (Table 2 and Table 3). The initial oleic acid content was consistent with that determined by Marquardt et al. [58], who reported 17 to 22% oleic acid in ghee produced from cattle–yak vs. yak breeds. It was observed that as the oleic decreased, its trans form, elaidic acid (Figure 7 and Figure 8), increased at a faster rate in FG0. There was a 47.92% and 13.13% increase in elaidic acid (main TFA present) in FG0 and GG0 after 30 min of MW-heating at 200 °C. During storage, the TFA slowly increased in both types of ghee, but its levels in FG0 increased sharply between 20 to 30 days (Figure 8). The elaidic acid contents of FG0 and GG0 increased by 62.03% and 27.02%, respectively, at the end of storage period. The increase in TFA during MW-heating/conventional heating has been recorded in dairy and edible oils even at low MW energy exposure. Topkafa and Ayyildiz [60] reported 7.27% TFA in corn oil that was MW-heated for 33.6 min. Afaneh et al. [61] observed a significant increase in the elaidic acid content when ghee was fried at 200 °C, but the elaidic acid content remained constant at 180 °C. Contrarily to this study, the non-variation in TFA at 180 °C could be due to conventional heating methods producing lower TFA levels than MW-heating [62]. Herzallah el al. [62] observed a 31% increase in TFA when milk was MW-heated for 5 min and a 16% increase after 30 min of conventional heating at 63 °C. 

The most abundant PUFA present in yak ghees were cis-9 and trans-11 conjugated linoleic acid (CLA) and alpha–linolenic acid (ALA). Other PUFA that were identified are shown in Figure 7 and Figure 8. In this study, the initial CLA and PUFA contents in yak ghee (Table 1 and Table 2) were higher than their contents in buffalo and cow ghee, where values of (1.6% and 2.6%) and (0.8% and 1.0%) were obtained [19]. This is consistent with a previous study that showed that yak butter had the highest PUFA levels and that its CLA content was almost four times higher than it was in cow, sheep, and goat butter [20]. The goji berry carotenoid enrichment of yak ghee using the “green” solvent extraction method used in this study did not cause any significant changes in the initial values of PUFA, CLA, and ALA; however, as MW-heating progressed, PUFA and CLA significantly decreased at a faster rate in FG0 than they did in GG0 (*p* < 0.05). At the highest temperature, MW-heating reduced the PUFA and CLA content in FG0 by 56.53% and 26.69%, respectively, while the PUFA and CLA in GG0 decreased by 39.45% and 20.54%, respectively, as ALA increased at 180 °C (15 min) (Table 2), but lower levels were recorded at increased MW-heating times and temperatures.

Under accelerated storage, the PUFA of FG0 and GG0 decreased by 66.01% and 44.06%, while CLA decreased to 68.89% and 55.78%, respectively, at the end of the storage period (Table 3). It was observed that the ALA of GG0 significantly increased from 0 to 20 days of storage, while that of FG0 only increased after 10 days and decreased in subsequent days (Figure 8). Previous studies have reported a decreasing effect of MW-heating on the PUFA and CLA of ghee and other edible oils. Ghee heated at 200 °C for 40 min lost 19.31% its PUFA content [63], while oil produced from peanut that was MW-heated at 170 °C for 9 h (320 W) lost 10.47% of its PUFA content [64]. Ghee stored at ambient temperature for 6 months lost 11.20% of its PUFA content. Aside from that, there was no significant difference between the MCFA contents of the FG0 and GG0 samples in the different MW-heating conditions, and their levels peaked at 180 °C (15 min) and slowly declined as the MW-heating temperature and duration increased (*p* < 0.05). SCFA, which were only butanoic and caproic acids was higher in FF0 than they were in GG0 and significantly increased with extensive MW-heating (Figure 7 and Table 2). SCFA and MCFA significantly increased during storage, with MCFA of both samples peaking after 10 days of storage (*p* < 0.05) (Table 3).

Changes in the fatty acids in yak ghee during MW-heating and accelerated storage was mainly due to tons of oxidation, isomerization, hydrolysis, and polymerization reactions that were severely influenced by factors such as the heating conditions and antioxidants present [2]. These numerous reactions led to the production of free fatty acids and partial- and polymerized glycerides, short chain-, trans-, conjugated-, epoxy-, hydroxyl-, keto-, and cyclic fatty acids [65]. UFA losses were due to oxidation reactions at their double bond sites. The higher the degree of unsaturation in fatty acids, the more susceptible they become to oxidation when exposed to promoters such as high temperature, light, prolonged storage, and MW energy. Generally, increases and decreases in the MUFA content is dependent on the oxidation rate of PUFA to MUFA and MUFA to SFA [59]. In this study, the enriched carotenoid content of GG0 enhanced RSA and thus protected the UFA and subsequently slowed down oxidation during heating and storage. The higher SFA in GG0 before treatments explains its higher initial oxidation stability (PV, TBA) compared to that of FG0. The high heating temperature and oxygen content are the major influencers of the isomerization of oleic acid into elaidic acid through the isomerization of the α—radicals of oleic acid that are produced from oxidation [2,66]. However, in the absence of oxygen or in cases where oxygen is limited, as in the case of the acceleration storage set up, elaidic acid was produced in small amounts in a minor oxidation pathway. Thus R_c_ radical forms R_t_ a radical that then reacts with the R_c_ H radical to make new R_c_ radicals, hence propagating the isomerization reaction. The accumulation of the excess of these free radicals as the antioxidants decreased resulted in an increased TFA content [66]. These mechanisms resulted in a continuous increase in elaidic acid in the yak ghee samples, with an increasing MW-heating temperature and time as well as its sharp rise in FG0 from 20 to 30 days of storage. According to Brühl [2], cis/trans isomerization is induced by excessive amounts of the 2-butene radical. The lower amount of TFA in GG0 than in FGO can be attributed to increased carotenoids inhibiting the isomerization of TFA from UFA [2]. Furthermore, the SCFA content increased during MW-heating and accelerated storage due to their increased formation from the various oxidative reactions that occurred in the ghee [2]. Some of the fatty acids in yak ghee are involved in biological functions that are beneficial to the human body. For instance, butyric acid induces the synthesis of epithelia mucin 2; MCFA promotes proper colon function; and oleic and CLA are involved in the prevention of cardiovascular diseases (CVD). However, palmitic, myristic, and elaidic acids have been linked with enhanced CVD occurrences [20,61]. 

## 4. Conclusions

Enrichment with goji berry carotenoids and the combination of the UAE and HSD technique greatly increased the total carotenoid content of yak ghee (180.85%). The goji berry carotenoids comparatively maintained the yellow color of yak ghee during storage, though it became darker during MW-heating. The carotenoid-enriched yak ghee had lower acid, peroxide, and thiobarbituric acid values but higher ABTS and DPPH values during microwave heating and accelerated storage. This signified that the carotenoid-enriched yak ghee had increased oxidative stability. Incorporating goji berry carotenoids into yak ghee did not have any significant effect on the initial values of conjugated linoleic acid and polyunsaturated fatty acids, but the saturated fatty acids increased, and the contents of the monounsaturated fatty acids and trans fatty acid decreased. However, during microwave heating and accelerated storage, there was a faster rate of unsaturated fatty acids loss, and the saturated fatty acids and trans fatty acids increased at a faster rate in FG0. These observations indicate that the goji carotenoids inhibited the oxidation of unsaturated fatty acids and suppressed the synthesis of the trans fatty acids in yak ghee during storage and microwave heating and hence conferred a protective effect on yak ghee. Therefore, the findings from this study support the use of goji berry carotenoids as a natural colorant and antioxidant in yak ghee. Additionally, in contrast to early observations, this study shows that yak ghee is beneficial to human health due to its richness in conjugated linoleic acid and other bioactive compounds, even after extensive storage and MW-heating. Future studies into sensory evaluation, phenolic content, and individual carotenoid contents in carotenoid-enriched yak ghee are recommended. 

## Figures and Tables

**Table 1:** Physical properties of yak ghee and goji berry carotenoid-enriched yak ghee before storage and heating. FG0 and GG0 represent raw yak ghee and goji berry carotenoid-enriched yak ghee.

Physical Properties		FG0	GG0
Moisture	%	0.25 ± 0.02	0.17 ± 0.01
Solids-not-fat (*w*/*w*)	%	0.00	1.08 ± 0.02
Fat Content	%	99.50 ± 0.48	98.6 ± 0.2
Yield	%	78.33 ± 1.33	81.15 ± 1.05

**Table 2:** Changes in the fatty acid groups of FG0 and GG0 during MW-heating. The labels 0, B15, B30, and D30 represent fatty acid values at zero and the MW temperatures 180 °C (15 and 30 min) and 200 °C for 30 min, respectively. “a, b, c, d” indicate significant differences within individual ghee treatments at *p* < 0.05. Asterisk (*) indicates significant differences between the two types of ghees at *p* < 0.05.

Fatty Acids%	FGO	GG0	FGB15	GGB15	FGB30	GGB30	FGD30	GGD30	Standard Deviations							
SCFA	0.96 ^c^*	0.90 ^c^*	0.99 ^c^	0.94 ^bc^	1.28 ^b^*	1.07 ^b^*	1.46 ^a^	1.24 ^a^	0.02	0.01	0.01	0.03	0.01	0.06	0.01	0.01
MCFA	4.14 ^b^	4.02 ^c^	6.00 ^a^	6.14 ^a^	5.99 ^a^	5.87 ^a^	5.47 ^a^*	5.21 ^b^*	0.13	0.05	0.17	0.26	0.21	0.18	0.26	0.21
SFA	58.48 ^d^*	60.50 ^c^*	61.80 ^c^*	62.84 ^b^*	65.51 ^b^*	63.50 ^b^*	66.05 ^a^*	64.33 ^a^*	0.45	0.51	0.39	0.48	0.71	0.66	0.53	0.69
MUFA	25.60 ^a^*	24.76 ^a^*	24.06 ^b^*	23.39 ^b^*	20.78 ^c^*	23.18 ^b^*	19.43 ^d^*	22.30 ^d^*	0.52	0.35	0.43	0.38	0.33	0.22	0.25	0.28
PUFA	8.12 ^a^	8.08 ^a^	5.73 ^b^*	6.91 ^b^*	4.01 ^c^*	6.07 ^c^*	3.53 ^d^*	5.15 ^d^*	0.16	0.44	0.20	0.15	0.18	0.25	0.20	0.10
TFA	8.24 ^d^*	6.25 ^d^*	8.78 ^c^*	6.91 ^c^*	9.92 ^b^*	7.20 ^b^*	11.18 ^a^*	7.88 ^a^*	0.18	0.22	0.06	0.11	0.13	0.18	0.19	0.14
Myristic	5.30 ^d^*	6.18 ^d^*	6.20 ^c^	6.51 ^c^	7.55 ^b^*	7.03 ^b^*	8.45 ^a^*	7.89 ^a^*	0.26	0.32	0.28	0.10	0.16	0.21	0.15	0.12
Palmitic	21.06 ^d^*	23.90 ^c^*	22.75 ^c^*	24.07 ^c^*	24.04 ^b^*	25.85 ^b^*	25.42 ^a^	26.18 ^a^*	0.09	0.16	0.37	0.30	0.20	0.20	0.29	0.23
Stearic	20.09 ^c^*	18.10 ^c^*	20.05 ^c^*	18.49 ^b^*	20.60 ^b^*	18.58 ^b^*	21.36 ^a^*	18.88 ^a^*	0.30	0.35	0.18	0.24	0.14	0.21	0.21	0.29
Oleic	20.59 ^a^*	19.24 ^a^*	19.02 ^b^	19.11 ^a^	17.50 ^c^*	18.95 ^b^*	16.64 ^d^*	18.35 ^c^*	0.50	0.41	0.15	0.26	0.32	0.18	0.00	0.05
Elaidic	6.19 ^c^*	4.96 ^c^*	6.83 ^c^*	5.00 ^c^*	8.21 ^b^*	5.21 ^b^*	9.16 ^a^*	5.60 ^a^*	0.07	0.10	0.00	0.01	0.02	0.04	0.10	0.06
ALA	1.47 ^b^	1.39 ^b^	1.51 ^a^*	1.73 ^a^*	0.90 ^c^*	1.24 ^c^*	0.75 ^d^*	1.00 ^d^*	0.01	0.03	0.01	0.01	0.01	0.02	0.01	0.01
**CLA**	2.81 ^a^	2.97 ^a^	2.68 ^b^*	2.92 ^a^*	2.44 ^c^*	2.83 ^b^*	2.06 ^d^*	2.36 ^c^*	0.04	0.01	0.01	0.02	0.02	0.01	0.01	0.01

**Table 3:** Changes in the fatty acid groups of FG0 and GG0 during accelerated storage. “a, b, c, d” indicate significant differences within individual ghee treatment at *p* < 0.05. Asterisk (*) indicates significant differences between the two types of ghees at *p* < 0.05. The labels 0, 10, 20, and 30 attached to FG and GG represent the storage days of raw yak ghee and goji berry carotenoid-enriched yak ghee.

Fatty Acids%	FGO	GG0	FG10	GG10	FG20	GG20	FG30	GG30	Standard Deviations							
SCFA	0.96 ^c^*	0.90 ^c^*	1.02 ^b^*	0.98 ^b^*	1.28 ^a^*	1.06 ^a^*	1.36 ^a^*	1.08 ^a^*	0.02	0.01	0.02	0.01	0.01	0.03	0.02	0.01
MCFA	4.14 ^b^	4.02 ^c^	4.54 ^ab^	4.66 ^a^	4.44 ^ab^*	4.08 ^c^*	4.75 ^a^*	4.34 ^b^*	0.13	0.05	0.20	0.26	0.11	0.15	0.24	0.32
SFA	58.48 ^d^*	60.50 ^c^*	61.58 ^b^	60.98 ^c^	66.34 ^b^*	63.21 ^b^*	69.59 ^a^*	66.71 ^a^*	0.45	0.51	0.57	0.49	0.60	0.73	0.55	0.67
MUFA	25.60 ^a^*	24.76 ^a^*	23.57 ^b^*	24.28 ^a^*	20.38 ^c^*	23.38 ^b^*	17.03 ^d^*	21.56 ^c^*	0.52	0.35	0.40	0.48	0.19	0.21	0.38	0.44
PUFA	8.12 ^a^	8.08 ^a^	6.81 ^b^*	7.66 ^b^*	4.03 ^c^*	6.57 ^c^*	2.76 ^d^*	4.52 ^d^*	0.16	0.44	0.25	0.15	0.35	0.21	0.27	0.30
TFA	8.24 ^d^*	6.25 ^d^*	8.56 ^c^*	6.81 ^bc^*	9.63 ^b^*	7.06 ^ab^*	11.03 ^a^*	7.30 ^a^*	0.18	0.22	0.30	0.35	0.19	0.27	0.20	0.23
Myristic	5.30 ^d^*	6.18 ^d^*	5.85 ^c^*	6.22 ^c^*	6.82 ^b^*	6.47 ^b^*	8.06 ^a^*	7.59 ^a^*	0.26	0.32	0.2	0.24	0.13	0.17	0.13	0.19
Palmitic	21.06 ^c^*	23.90 ^d^*	23.00 ^b^*	24.32 ^c^*	24.99 ^a^*	25.53 ^b^*	25.29 ^a^*	26.43 ^a^*	0.09	0.16	0.33	0.26	0.20	0.23	0.26	0.21
Stearic	20.09 ^c^*	18.10 ^c^*	20.30 ^c^*	18.47 ^bc^*	21.00 ^b^*	18.88 ^b^*	21.80 ^a^*	19.63 ^a^*	0.30	0.35	0.25	0.11	0.17	0.21	0.22	0.31
Oleic	20.59 ^a^*	19.24 ^a^*	18.70 ^b^*	19.01 ^b^*	15.92 ^c^*	18.41 ^c^*	13.07 ^d^*	17.12 ^d^*	0.50	0.41	0.31	0.23	0.30	0.18	0.19	0.15
Elaidic	6.19 ^d^*	4.96 ^c^*	6.86 ^c^*	5.61 ^b^*	8.63 ^b^*	5.83 ^b^*	10.03 ^a^*	6.30 ^a^*	0.07	0.10	0.05	0.04	0.03	0.02	0.11	0.10
ALA	1.47 ^a^	1.39 ^b^	1.49 ^a^	1.42 ^b^	1.03 ^b^*	1.61 ^a^*	0.64 ^c^*	1.07 ^c^*	0.01	0.03	0.01	0.01	0.01	0.01	0.01	0.02
CLA	2.81 ^a^	2.97 ^a^	2.30 ^b^*	2.86 ^a^*	1.96 ^c^*	2.58 ^b^*	1.54 ^d^*	2.05 ^c^*	0.04	0.01	0.01	0.01	0.01	0.02	0.01	0.01

## Data Availability

The data presented in this study are available upon request from the corresponding author. The data are not publicly available due to privacy.

## Abbreviations

FG0	Fresh yak ghee at 0
GG0	Goji berry carotenoid-enriched yak ghee
FGB15	Fresh yak ghee heated at 180 °C for 15 min
FGB30v	Fresh ghee heated at 180 °C for 30 min
GGB15	Goji berry ghee heated at 180 °C for 15 min
GGB30	Goji berry ghee heated at 180 °C for 30 min
FGD30	Goji berry ghee heated at 200 °C for 30 min
FG10	Fresh yak ghee stored for 10 days
FG20	Fresh yak ghee stored for 20 days
FG30	Fresh yak ghee stored for 30 days
GG10	Goji berry yak ghee stored for 10 days
GG20	Goji berry yak ghee stored for 20 days
GG30	Goji berry yak ghee stored for 30 days
UAE	Ultrasound-assisted extraction
HSD	High shear dispersion
PUFA	Poly unsaturated fatty acids
TFA	Trans fatty acids
MUFA	Monounsaturated fatty acids
SFA	Saturated fatty acids
CLA	Conjugated linoleic acid
TCC	Total carotenoid content
SCFA	Sort chain fatty acids
MCFA	Medium chain fatty acids
ABTS	2,2′-Azinobis(3-ethylbenzothiazoline-6-sulfonic acid)
DPPH	2,2-diphenyl-1-picrylhydrazyl
AV	Acid value
PV	Peroxide value
TBA	Thiobarbituric acid
